# CNN-Based Multimodal Human Recognition in Surveillance Environments

**DOI:** 10.3390/s18093040

**Published:** 2018-09-11

**Authors:** Ja Hyung Koo, Se Woon Cho, Na Rae Baek, Min Cheol Kim, Kang Ryoung Park

**Affiliations:** Division of Electronics and Electrical Engineering, Dongguk University, 30 Pil-dong-ro, 1-gil, Jung-gu, Seoul 100-715, Korea; koo6190@dongguk.edu (J.H.K.); jsu319@dongguk.edu (S.W.C.); naris27@dongguk.edu (N.R.B.); mincheolkim@dongguk.edu (M.C.K.)

**Keywords:** multimodal human recognition, surveillance environment, CNN, human recognition by face and body

## Abstract

In the current field of human recognition, most of the research being performed currently is focused on re-identification of different body images taken by several cameras in an outdoor environment. On the other hand, there is almost no research being performed on indoor human recognition. Previous research on indoor recognition has mainly focused on face recognition because the camera is usually closer to a person in an indoor environment than an outdoor environment. However, due to the nature of indoor surveillance cameras, which are installed near the ceiling and capture images from above in a downward direction, people do not look directly at the cameras in most cases. Thus, it is often difficult to capture front face images, and when this is the case, facial recognition accuracy is greatly reduced. To overcome this problem, we can consider using the face and body for human recognition. However, when images are captured by indoor cameras rather than outdoor cameras, in many cases only part of the target body is included in the camera viewing angle and only part of the body is captured, which reduces the accuracy of human recognition. To address all of these problems, this paper proposes a multimodal human recognition method that uses both the face and body and is based on a deep convolutional neural network (CNN). Specifically, to solve the problem of not capturing part of the body, the results of recognizing the face and body through separate CNNs of VGG Face-16 and ResNet-50 are combined based on the score-level fusion by Weighted Sum rule to improve recognition performance. The results of experiments conducted using the custom-made Dongguk face and body database (DFB-DB1) and the open ChokePoint database demonstrate that the method proposed in this study achieves high recognition accuracy (the equal error rates of 1.52% and 0.58%, respectively) in comparison to face or body single modality-based recognition and other methods used in previous studies.

## 1. Introduction

Previous biometrics studies have used various modalities, including the face, fingerprints, body, irises, retinas veins, and voice [1,2,3,4,5,6,7,8,9]. In a typical surveillance camera environment, it is difficult to use fingerprints or vein recognition, so face, body, and iris methods have been considered. In the case of iris recognition, a zoom lens and a near-infrared (NIR) light illuminator of high power are needed to capture iris images at a distance, so the systems are large and expensive and can be used in a limited range of contexts. Also, in a surveillance environment, the camera is normally installed above the user and captures images in a downward direction, so it mainly takes off-angle images that capture the user’s iris at an angle. In such circumstances, the recognition accuracy is greatly reduced [9].

Face recognition has often been considered for surveillance environments as it can generally be conducted in a visible light camera environment. However, in a surveillance environment, most cases involve a camera capturing images in a downward direction from above and people do not look directly at the camera. Thus, it is generally difficult to capture front facial images, and in such cases, facial recognition accuracy is greatly reduced. To address this issue, 105 composite geometrical descriptors for 3D face analysis based on 3D face data captured by a laser scanner were presented in a previous study [10]. The authors mapped these new descriptors on 217 facial depth maps and analysed them based on descriptiveness of facial shape and exploitability for detecting landmark points. In other research [11], they proposed a method to automatically diagnose and formalize prenatal cleft lip with key points and recognize the type of defect in 3D ultrasonography. For that, they adopted differential geometry as a framework to describe facial curvatures and shapes. In previous research [12], Cowie, et al. introduced the various methods of emotion recognition in human-computer interaction including applications, framework, input and output-related issues, physiological and domain issues, training and test materials, and case study, etc. In [13], Tsapatsoulis et al. proposed the method of face extraction from non-uniform background based on the fusion of a retrainable neural network and morphological size distribution method. In addition, they also proposed the face recognition in MPEG-4 compressed domain to fuse the face images of high quality and low computational complexity.

In this research, we consider using face and body data for human recognition in a visible light camera surveillance environment, which is based on the movements of people’s bodies and the texture, color, and shape of their bodies. However, when images are captured by a camera installed in an indoor surveillance environment, in many cases, part of the target body is not included in the camera viewing angle, and only part of the body is captured, which causes a drop in human recognition accuracy. Aside from this, a study was conducted on human recognition using body images captured by visible light and thermal cameras [14], but this method requires high-cost thermal cameras, so it is not suitable for use in a normal surveillance environment. The next section analyzes previous studies on human recognition using face and body data in a surveillance camera environment.

## 2. Related Work

Previous studies on human recognition in a surveillance environment using face and body data can be broadly divided into single-modality-based methods and multiple-modality-based methods. The former include face recognition, movement-based body recognition, as well as texture-, color-, and shape-based body recognition. In a study on face recognition in a surveillance camera environment, Kamgar-Parsi et al. detected face regions through the boosted classifier of Haar wavelets method and performed morphing of facial images based on an active shape model (ASM), followed by chi-square distribution-based classification [15]. An et al. used several cameras to capture face images and performed face recognition based on a dynamic Bayesian network (DBN) [16]. Grgic et al. installed five cameras above a door and captured face data at three set locations to perform face recognition using a principal component analysis (PCA) method [17]. Banerjee et al. performed recognition using a soft-margin-based learning method for multiple feature-kernel combinations (SML-MKFC) with domain adaptation (DA) [18]. In this type of surveillance environment, it is often difficult to capture a front face image, hence, the face recognition accuracy is reduced. To address this problem, there have been studies [19] on recognition by extracting landmark points on a face and using these to adjust the face to a front angle (face frontalization). However, due to the nature of surveillance camera environments (especially indoor environments), in many cases, the face region is subject to optical and motion blurring due to the target moving at a short distance from the camera. In such cases, facial landmark point extraction is not precise, so face frontalization cannot be performed. Therefore, recognition methods have been developed that use the body region’s texture, color, and shape information obtained from a single image, as well as methods that use body motion from several continuous images. Methods of the former kind include the following.

Though not focused on human identification, Antipov et al. compared the performance of hand-crafted feature-based person re-identification and a histogram of oriented gradients with the performance of learned features that were based on a mini-convolutional neural network (CNN) and AlexNet-CNN for the purpose of gender recognition [20]. Layne et al. studied body image-based recognition using a method of symmetry-driven accumulation of local features (SDALF) with metric learning attributes (MLA) [21]. Nguyen et al. performed a gender recognition study using histogram of oriented gradient (HOG), PCA, and support vector machine (SVM) on user body images captured by visible light and thermal cameras [22]. Also, in [14], AlexNet and PCA-based feature extraction and distance measurement were used to perform a study on personal identification based on user body images captured by visible light and thermal cameras. Figueira et al. performed a study on person re-identification based on a semi-supervised multi-feature learning (MFL) method [23]. In [24], a method of person re-identification was proposed that uses spatial covariance regions of human body parts and spatial pyramid matching. Prosser et al. used a Gabor and Schmid filter to perform feature extraction and then used ensemble ranking SVM to perform person re-identification [25]. Ensemble ranking SVM was proposed as a method to overcome the scalability limitation of existing SVM-based ranking problems. Chen et al. used a spatially constrained similarity function on a polynomial feature map (SCSP) and PCA to perform feature extraction and performed a study on person re-identification based on spatial pyramid matching [26]. Liao et al. performed a study on person re-identification based on local maximal occurrence (LOMO) and cross-view quadratic discriminant analysis (XQDA) [27]. In [28], person re-identification was performed using a filter pairing neural network (FPNN) to resolve the problems of misalignment, photometric and geometric transforms, occlusions, and background clutter. In both [29,30] a Siamese CNN (S-CNN) structure was used for person re-identification, but the methods were different in that [29] used 7 convolution blocks, whereas [30] used two convolutional layers. In [31], a positive mining method was proposed for training a CNN for person re-identification, and discriminative deep metric learning (DDML) was applied. Yang et al. proposed training multi-level (e.g., pixel-level, patch-level, and image-level) descriptors using weighted linear coding (WLC) for person re-identification [32].

In these ways, recognition methods that use texture, color, and shape data from body regions obtained from a single image can compensate for their face recognition disadvantages, but they still have the disadvantage that they can misidentify an imposter as being the genuine person if they wear clothes of a similar color or arrangement. They can also show degraded recognition performance when part of the target body is not captured in an image. To resolve this problem, recognition methods have been proposed that use body motion and so forth in several continuous images. In [33], a study was performed on PCA and silhouette analysis-based gait recognition for human identification. In [34], gait-based recognition was further investigated, but to resolve the problem of insufficient existing gait data, synthetic gait energy images (GEI) were obtained, and the synthetic GEI and the features extracted from PCA and multiple discriminant analysis (MDA) were combined and was applied to improve recognition performance. However, these studies have the disadvantage that they can mainly be used with continuous images of a person’s side (images in which the person is moving perpendicular to the direction from which the camera is shooting), but it is difficult to use them when the person approaches or moves further away from the camera.

In view of these problems, multimodal human recognition methods that combine face and body data have been proposed, and these previous methods mainly combine side face recognition and movement-based body recognition. In [35,36], feature extraction was performed using enhanced side-face images (ESFI) and the GEI method. Then, PCA and MDA were performed and recognition was done through a fusion method based on sum, product, and max rules. In [37], side face and GEI features were converted using PCA and MDA, respectively, and combined at the feature level to perform recognition. In [38], side face recognition was done through curvature-based matching, and gait recognition was done through direct GEI matching. Then the two types of recognition data were combined through a sum rule and a product rule. Kale et al. used posterior distribution and template matching methods for gait and side face recognition, and they performed fusion through sum, min, and product rules [39]. 

In [44], a study on face and gait recognition using image-based visual hull (VH) was performed. In [45], view-normalized sequences were used to perform gait recognition and face recognition, and they were combined through a cross-modal fusion rule to improve recognition performance. Guan et al. performed a study in which face recognition based on kernel Fisher analysis (KFA) was combined with gait recognition based on a random subspace method (RSM) [46]. Hofmann et al. used the eigenface calculation and α-GEI methods for a combined recognition of face and gait, respectively, in the Human ID Gait Challenge [47]. Liu et al. performed a study on the combined recognition of face and gait based on a hidden Markov model (HMM) and Gabor feature-based elastic bunch graph matching (EBGM) methods [48]. Also, Geng et al. performed a study on distance-driven fusion of face recognition, which was based on Fisherface, and gait recognition, which was based on silhouette image-based locality preserving projection (LPP) [49]. Most of these methods were applied to continuous images of a person’s side (images in which the person is moving perpendicularly to the camera’s shooting direction), and these methods have the disadvantage of being difficult to apply when the person is approaching or moving further away from the camera. In addition, they must process several continuous images, so they also have the drawback of a long processing time. To resolve these problems, this paper presents a deep CNN-based multimodal human recognition method that uses both face and body data in a single image. In addition, this method can be used to perform recognition in cases where the person is approaching or moving further away from the camera. These cases occur frequently in indoor surveillance (especially hallway) environments, but they were not sufficiently addressed by previous studies. Table 1 shows the advantages and disadvantages of the methods proposed in previous studies on human recognition in a surveillance camera environment and present study.

## 3. Contribution of Our Research

Our research is novel in the following four ways in comparison to previous works:

**–**  Previous methods for face- and body-based multimodal human recognition have mainly been based on continuous images of the side face and gait captured during lateral movement relative to the camera. However, this study focuses on cases that often occur in indoor surveillance camera environments (especially hallways) in which a person is approaching or moving further away from the camera; the proposed method is the first approach for the multimodal human recognition that separately recognizes face and body regions in a single image and combines them.
**–**  The person’s whole body image is not used as a single CNN input. Rather, the face region and the body region are separated, and each is used as a separate CNN input. Thus, more detailed texture, color, and shape information regarding each region can be used. As a result, the recognition accuracy can be improved beyond that of methods that use whole body images as a single CNN input.
**–**  A visual geometry group (VGG) Face-16 CNN is used for the face region, and a residual network (ResNet)-50 CNN is used for the body region. The body region is larger than the face region, and more detailed texture, color, and shape data must be extracted from the clothes and body. Therefore, the ResNet-50 is used because it has more layers and uses detailed residual information. On the other hand, the face region is smaller than the body region, and recognition normally uses more mid- or low-frequency information than high-frequency information, so the VGG Face-16 is used rather than the ResNet-50, which uses detailed residual information.
**–**  Unlike previous methods that only focus on cases in which the entire body is included in the input image, the targets of the proposed method also include images in which part of the body region cannot be seen in the input image. To make impartial comparison experiments possible, the Dongguk face and body database (DFB-DB1), which was custom made using two kinds of cameras to evaluate performance in a variety of camera environments, and the VGG Face-16 and ResNet-50 CNN models were made public to other researchers in [53].

## 4. Proposed Method

### 4.1. Overall Procedure of Proposed Method

**Figure 1:**
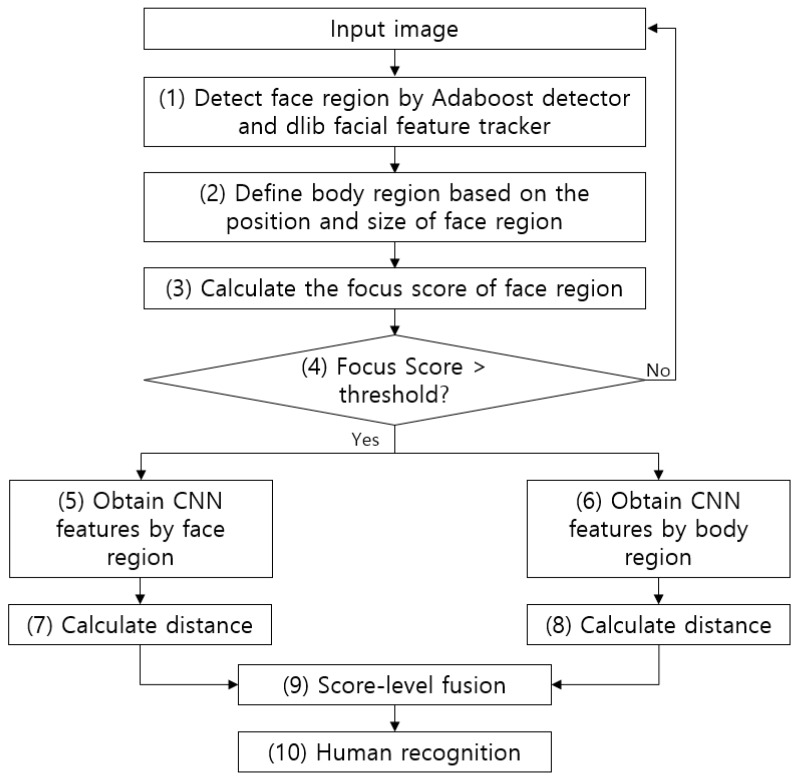
Overall procedure of proposed method.

Figure 1 shows an overall flowchart of the proposed method. First, the face region in an image captured by a surveillance camera is detected by the adaptive boosting (AdaBoost) detector [54]. Then, a more accurate face region is detected based on the positions of the facial features (both eyes) detected by the dlib facial feature tracker [55] (step (1) in Figure 1). After this, the body region is defined based on the position and size of the detected face region (step (2) in Figure 1). In the next step, the face region’s focus score is measured and the next recognition step is only performed if this value is above a certain threshold (steps (3) and (4) in Figure 1). If it is not, the next image is acquired from the camera. After this, the CNN models are run using the face region and body region as separate inputs (steps (5) and (6) in Figure 1). The extracted CNN features are used to measure their distance from the already registered features (steps (7) and (8) in Figure 1). Score-level fusion is performed using the two obtained distances, and a final matching score is obtained. This is then used to perform human recognition (steps (9) and (10) in Figure 1).

### 4.2. Detection of Face and Body Regions as well as Focus Measurement

**Figure 2:**
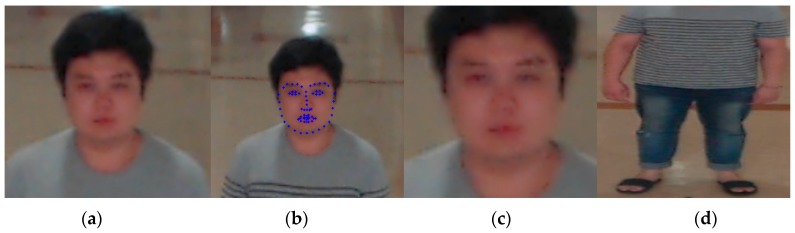
Detection of face and body region. (**a**) Face region detected by AdaBoost algorithm from input image, (**b**) facial landmarks detected in face region by dlib facial feature tracker, (**c**) redefined face region based on eye landmarks, (**d**) defined body region.

As explained in Section 4.1 and shown in Figure 2, the AdaBoost detector is used to detect the face region in an image captured by a camera [54]. AdaBoost detector uses the cascaded weak classifiers based on Haar feature, and it has been widely used for face detection. In this research, we used the AdaBoost detector provided from OpenCV library [56] without additional training with our experimental images. AdaBoost detector can generate the roughly detected face box which includes face and the part of background. Therefore, a more accurate face region is detected based on the positions of facial features (both eyes) detected by the dlib facial feature tracker [55]. In this research, we used the open source of dlib facial feature tracker provided from [55] without additional training with our experimental images. Also, as explained in Section 4.1, the body region is defined based on the size and position of the detected face region and anthropometric data on a normal person’s body, as shown in Figure 2d. In details, based on the center position (*x__face_*, *y__face_*), width (*w__face_*), and height (*h__face_*) of the detected face region, the center position (*x__face_*, *y__face_* + 1.8 × *h__face_*), width (1.8 × *w__face_*), and height (2.2 × *h__face_*) of body box are defined, respectively. The lowest vertical position of body box is limited by “image height—1”. In addition, the left- and right-most positions of body box are limited by “0” and “image width—1”, respectively.

**Figure 3:**
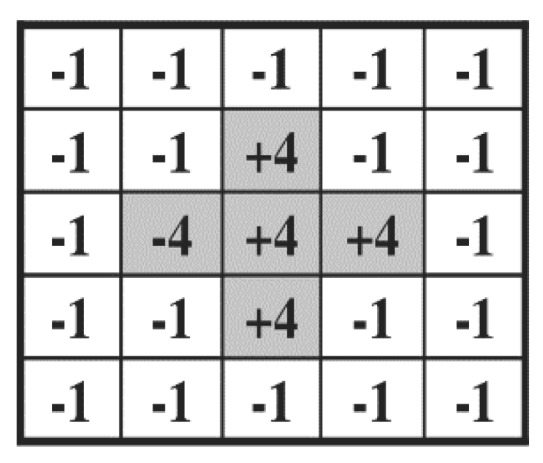
The 5 × 5 mask for focus assessment.

After this, the 5 × 5 mask proposed in [57] is used on the face region to calculate the focus score. The shape of this mask is shown in Figure 3. The 5 × 5 mask was designed to measure the amount of high frequency component in image [57]. In details, the magnitude value is computed by the convolution operation with the 5 × 5 convolution kernel in the image based on the moving step of 1 pixel both in horizontal and vertical directions as shown in Equations (1) and (2). Then, this magnitude value (FS of Equation (2)) is normalized so as to be presented in the range from 0 to 100 based on min-max scaling, and min and max values were determined from the training data. This normalized value is used as final focus score, and the higher the score, the better the focus condition. The next recognition step is only performed if this focus score is above a certain threshold. If it is not, the next image is acquired from the camera instead of recognition. The optimal threshold of focus score was experimentally determined as 20 (both in DFB-DB1 and ChokePoint databases) from the training data so as to obtain the highest accuracy of recognition.

(1) $$O\left[x,y\right]=I\left[x,y\right]‗M\left[x,y\right]=\sum_{q=0}^{H-1}\sum_{p=0}^{W-1}I\left[p,q\right]\;M\left[x-p,y-q\right]$$

(2) $$\mathrm{FS}=(\sum_{y=0}^{H-1}\sum_{x=0}^{W-1}O\left[x,y\right])/\left(W\times H\right)$$

**Figure 4:**
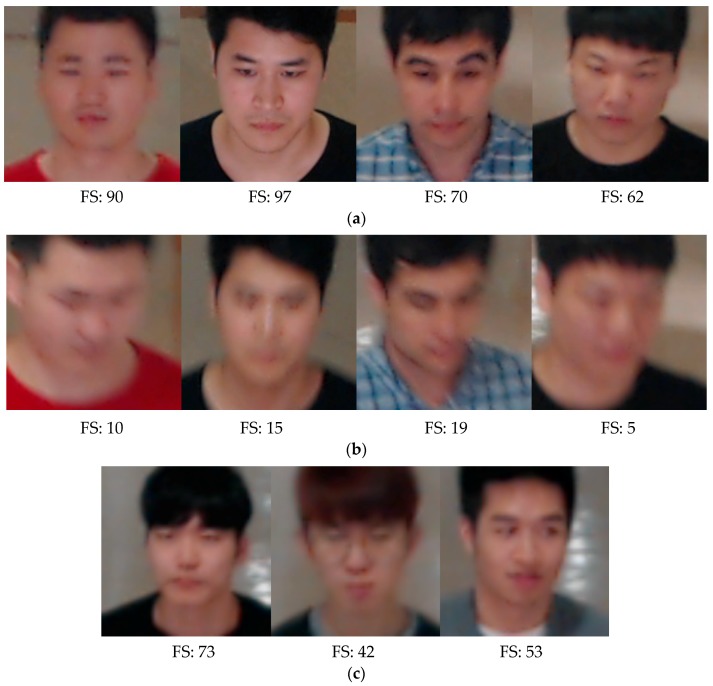
DFB-DB focus score calculation example images: (**a**) Examples of images with a focus score (FS) above the threshold (20) (BCC950 camera images). (**b**) Examples of images with a focus score below the threshold (BCC950 camera images). (**c**) Examples of images with a focus score above the threshold (20) (C920 camera images).

**Figure 5:**
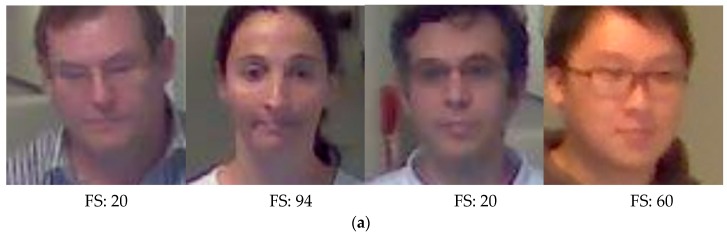

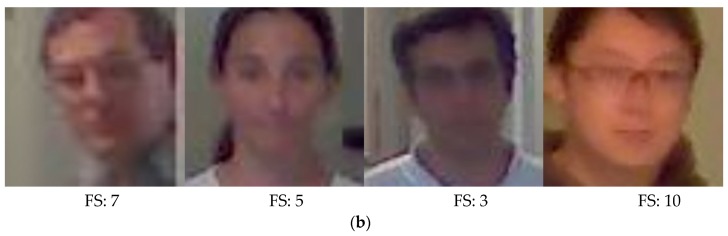
ChokePoint dataset focus score calculation example images: (**a**) Examples of images with a focus score (FS) above the threshold (20). (**b**) Examples of images with a focus score below the threshold.

In Equations (1) and (2), I[x,y], O[x,y] and M[x,y] are input, output, and 5 × 5 mask images, respectively. *W* and *H* are the image width and height, respectively. In the DFB-DB1 database, which was custom made for this study, images were captured by two types of cameras, namely, the Logitech BCC950 [58] and the Logitech C920 [59], to evaluate performance of the proposed method in a variety of camera environments. Figure 4 shows the focus scores of images in DFB-DB1. Also, Figure 5 shows the focus scores of the ChokePoint dataset [60], which is an open database used in this study. As seen by a comparison of Figure 4b and Figure 5b, the blurring due to user movement is more severe in the images in Figure 4b.

### 4.3. CNN for Face Recognition

**Figure 6:**
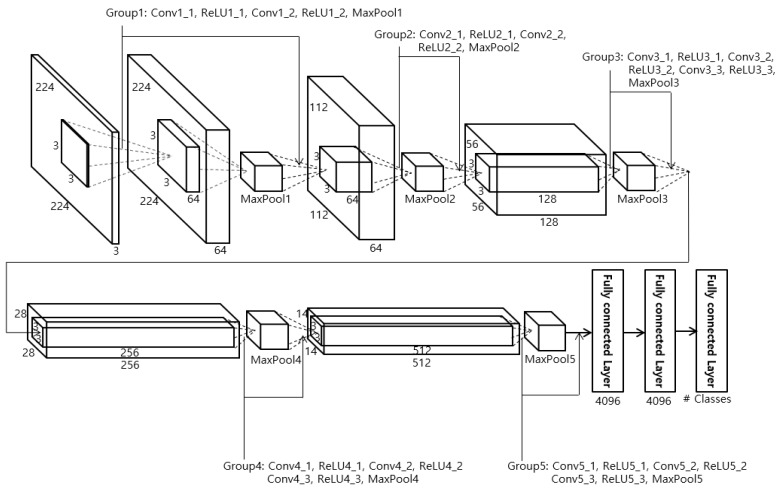
The structure of VGG Face-16 [3]. Conv, ReLU, and MaxPool represent convolutional layer, rectified linear unit layer, and max pooling layer, respectively.

In the proposed method, face recognition is performed using the VGG Face-16 CNN model, which takes the facial regions obtained in Section 4.2 as input. The VGG Face-16 CNN model is used for the face region, and the ResNet-50 CNN model is used for the body region. We used the VGG Face-16 CNN model provided from [61] in this research. The body region is larger than the face region, and more detailed texture, color, and shape data must be extracted from the clothes and body, so the ResNet-50 is used, as it has more layers and detailed residual information. On the other hand, the face region is smaller than the body region, and recognition normally uses more mid- or low-frequency information than high-frequency information, so the VGG Face-16 is used rather than the ResNet-50, which uses detailed residual information. To fine-tune the pre-trained VGG Face-16 model [3] with the database used in this study, the detected face regions from Section 4.2 are normalized to a 224 × 224 pixels size. The normalization was performed by bi-linear interpolation. VGG Face-16 has the same structure as VGG Net-16 with 13 convolutional layers, 5 pooling layers, and 3 fully connected layers, as shown in Figure 6 and Table 2. VGG Face-16 and VGG Net-16 have no structural differences, but they were trained differently. That is, VGG Face-16 is a model trained with labeled faces in the wild [62] and YouTube faces [63], while VGG Net-16 [64] is a model trained in the ImageNet large-scale visual recognition competition (ILSVRC)-2014 [65]. Normally, the size of a feature map obtained from the convolution operation in a CNN is calculated from the width or height of the filter, the width or height of the input image (or feature map) before it enters the convolutional layer, the amount of padding in the convolutional layer, and the number of strides [66]. After passing through the convolution layer, the rectified linear unit (ReLU) layer [67] is next. Normally, non-overlapping pooling windows obtain better results [68], so a filter size of 2 × 2 with a stride of 2 × 2 was used in this study. The final layer is the fully connected layer (FCL). In the 3rd FCL, there is a softmax layer. Finally, to avoid overfitting in the training data used during fine-tuning, dropout layers are used in the 1st and 2nd FCLs. In this study, the dropout layer probability was set at 50%.

### 4.4. CNN for Human Recognition Using Body

**Figure 7:**
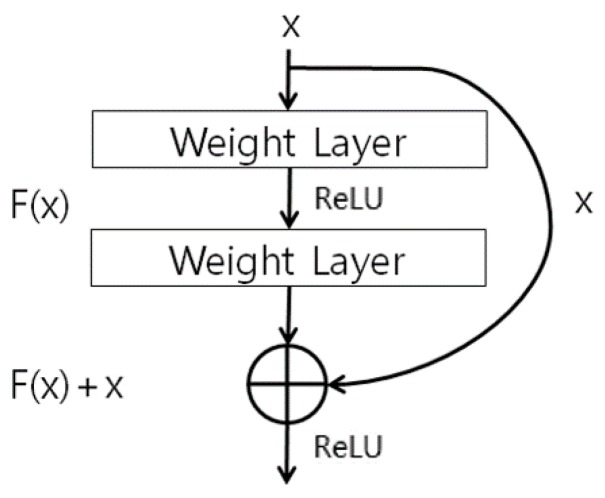
Shortcut for residual learning in ResNet. ReLU means rectified linear unit layer.

**Figure 8:**
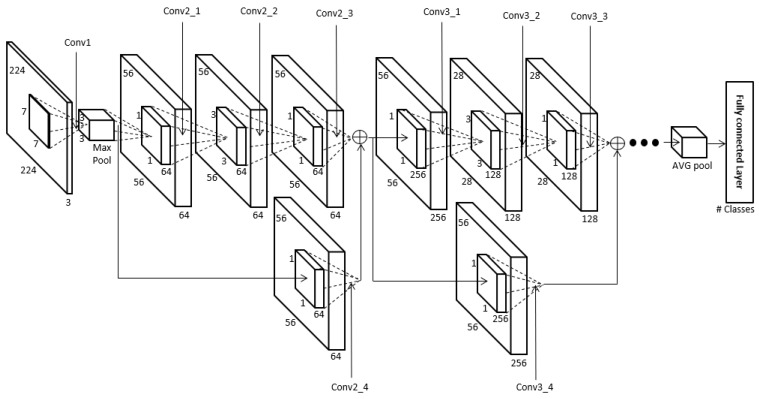
The structure of ResNet-50 [70]. Conv, MaxPool, and AVG pool represent convolutional layer, max pooling layer, and average pooling layer, respectively.

The body region obtained in Section 4.2 is used as input for the ResNet-50 CNN to perform human recognition using body data. In this research we used the ResNet-50 CNN model provided in [69]. One of the ResNet-50 model’s main features is the shortcut structure for residual learning shown in Figure 7 [70]. ResNet has many convolutional layers, so the feature map size becomes smaller the farther back one goes, and the vanishing or exploding gradient problem occurs as the feature map’s feature values become smaller. Therefore, the shortcut structure shown in Figure 7 is used. Also, ResNet forms a bottleneck structure. The reason for this is that using 1 × 1, 3 × 3, and 1 × 1 convolutions rather than two 3 × 3 convolutions can reduce the computation time [70]. Batch normalization is performed before activation function and after each convolution [70,71]. In this study, the pre-trained ResNet-50 was fine-tuned with the training data. This ResNet-50 structure is shown in Figure 8 and Table 3.

### 4.5. Training of CNN Model by Stochastic Gradient Descent Method

The stochastic gradient descent (SGD) method was used to train the VGG Face-16 and ResNet-50 used in this paper. SGD is a type of gradient descent method, and it is expressed as [72]:

(3) $$W_{n+1}=W_{n}-\gamma \nabla F\left(W_{n}\right),$$

where *W* represents the parameters of the CNN which must be found via training. It consists of the product of the movement distance γ from the activation function F(*x*), which takes the value of the previous parameters as input. Depending on whether the initial starting point is a negative number or positive number, γ∇F(x) amount of movement is made in the opposite direction. Unlike the gradient descent (GD) method, which uses all the training data to find the optimal parameters, in the SGD method training is performed in mini-batch units (*Z* of Equation (4)) randomly selected from the overall training data [72]:

(4) $$W_{n+1}=W_{n}-\gamma \nabla F\left(Z_{n},W_{n}\right).$$

The codes of SGD method for VGG Face-16 and ResNet-50 are provided from [61,69], respectively. The detail parameters for SGD method used in our experiments are explained in Section 5.2.

### 4.6. Calculation of Distance and Score-Level Fusion

In the next step, the 4096 features behind the 2nd fully connected layer in Table 2 are used as features for face recognition, and the 2048 features behind the AVG pool in Table 3 are used as features for human recognition using body. After this, we find each of the Euclidean distances from the features previously extracted from the enrolled images. The two Euclidean distances are normalized through min–max scaling, and score-level fusion is performed to find the final matching score. Here, the min and max values for min–max scaling are found in the training data. For score-level fusion, the weighted sum and weighted product rules are used. For score level fusion, two scores from face and human recognition using body are normalized via min-max scaling, and optimal weights for score level fusion were found from the training data. Based on the fused score, recognition is performed. In detail, in case of verification (1:1 matching), if the fused score is less than the predetermined threshold, the input image is accepted as genuine matching. If not, it is rejected as imposter matching. Here, the genuine matching means the case that input and enrolled images are from a same class whereas the imposter matching represents the case that input and enrolled images are from a different class. The optimal threshold was experimentally determined with training data so as to obtain the minimum equal error rate (EER) of recognition. There are two types of error rates such as false acceptance rate (FAR) and false rejection rate (FRR). These two error rates have the trade-off relationship. That is, the larger the FAR, the smaller the FRR. The EER is the error rate when FAR is same to FRR. In case of identification (1:n matching), one enrolled image (among n images) which shows the smallest fused score with the input is determined as that of same class to the input image.

## 5. Experimental Results and Analysis

### 5.1. Experimental Data and Environment

**Figure 9:**
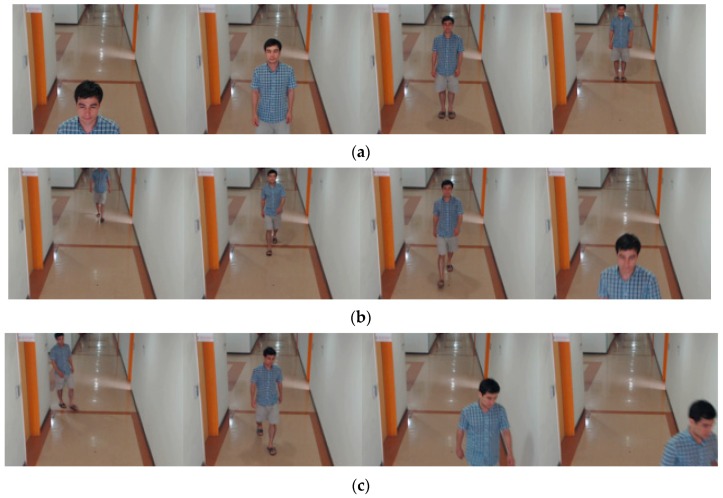
Example images from DFB-DB1 taken by the Logitech BCC 950 camera. (**a**) One-person still image. (**b**) One-person straight-line movement image. (**c**) One-person corner movement image.

**Figure 10:**
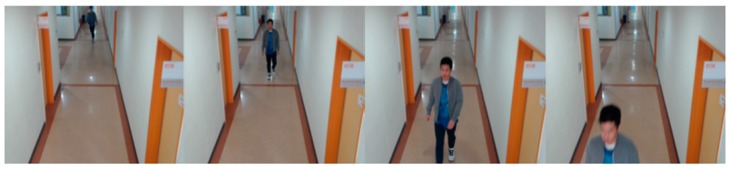
Example images from DFB-DB1 taken by the Logitech C920 camera.

In this study, DFB-DB1 was created for the experiments using images of 22 people obtained by two types of cameras to assess the performance of the proposed method in a variety of camera environments. The first camera was a Logitech BCC 950 [58], and the camera specifications include a camera viewing angle of 78°, a maximum resolution of full high-definition (HD) 1080 p, and auto-focusing at 30 frames per second (fps). The second camera was a Logitech C920 [59], and its specifications include a maximum resolution of full HD 1080p, a viewing angle of 78° at 30 fps, and auto focusing. Images were taken in an indoor environment with indoor lights on, and each camera was installed at a height of 2 m 40 cm. Before collecting DFB-DB1, we gave the sufficient explanations of our experiments to acquire DFB-DB1 to all the participants. In addition, we obtained the informed and signed consent forms from all the participants before collecting DFB-DB1, and all the participants also agreed to show their faces and bodies (without any pre-processing) in our paper. The database was divided into two categories according to the camera. In the first database, the images were captured by the Logitech BCC 950 based on the scenarios of one, two, and three people, including the images of two cases where the target body was still and when it was moving. The still images were captured in four positions, and the moving images were divided into two cases (straight-line movement and corner movement) and captured. We requested all the participants to move naturally without noticing the situation of collecting our DFB-DB1, and did our best for collecting DFB-DB1 in the real-world scenario. Examples of still images and movement images are shown in Figure 9. The second database is composed of the images obtained by the Logitech C920, and the angle of camera was similar to that for capturing the first database. In the second database, the images were captured based on the scenario of 1 people and the case where the target body was moving (straight-line movement) by three times, as shown in Figure 10.

Table 4 contains a description of DFB-DB1. This study executed a two-fold cross validation scheme, so DFB-DB1 was divided into sub-databases 1 and 2. In the first cross validation, sub-database 1 was used for training and sub-database 2 was used for testing. In the 2nd fold cross validation, sub-database 2 was used for training, and sub-database 1 was used for testing. Sub-databases 1 and 2 were made to contain images of different people. Also, DFB-DB1 and the VGG Face-16 and ResNet-50 models which were trained in this study were made public for other researchers in [53] so that impartial comparison experiments could be performed.

**Figure 11:**
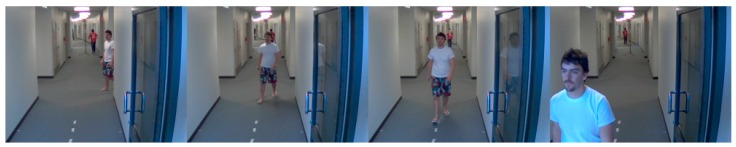
ChokePoint dataset image example.

The ChokePoint database is a real-world surveillance video database which was designed for person identification and verification experiments and is provided by National ICT Australia Ltd. (NICTA) as an open database [60]. It consists of Portals 1 and 2. Portal 1 contains images of 25 people (19 males and 6 females), and Portal 2 contains images of 29 people (23 males and six females). Portals 1 and 2 were captured during a one-month time span. The images for each location were captured with three cameras, and at a total of six locations. In this study, the location P2L was selected from among the six locations as it is similar to the location in the DFB-DB images. As previously mentioned, the P2L database contains images of a total of 29 people. In this study 28 people were selected for two-fold cross validation. Fourteen classes were set for each of the sub-databases 1 and 2. Examples from the ChokePoint database are shown in Figure 11, and descriptions of the ChokePoint database are provided in Table 4.

In this study, the training and tests were performed in a desktop environment that included an Intel Core i7-6700 CPU @ 3.4 GHz (four cores) with 16 GB of RAM, and NVIDIA GeForce GTX 1070 with a graphics memory of 8 GB [73] (CUDA 8.0). The Windows Caffe framework (version 1) [74], Microsoft Visual Studio 2013 [75], and OpenCV library (ver. 2.4.10) [56] were used to implement the algorithm.

### 5.2. Training of CNN Model

To resolve the problem of the CNN not receiving adequate training due to insufficient training data, training in this study was performed using data that was increased through the augmentation of the training data using the method described below. As shown in Table 4, data augmentation was performed only on the training data, and only unaugmented original data was used for the testing data.

**Figure 12:**
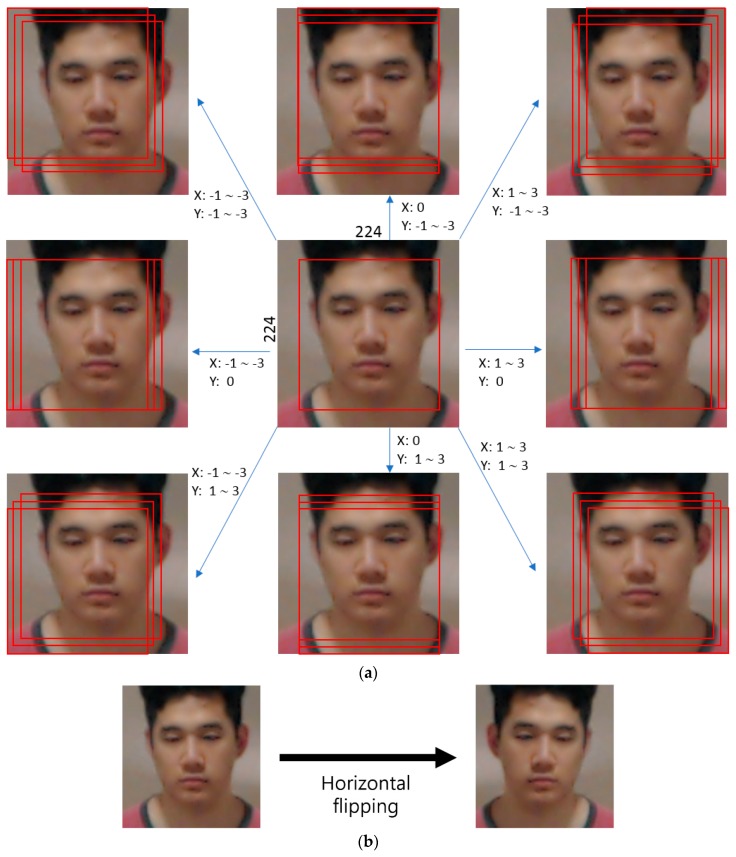
Data augmentation method by (**a**) image translation and cropping, and (**b**) horizontal flipping.

In DFB-DB1, the number of images for each class (person) is different, so when augmentation was performed, classes with over 100 images underwent a process of 3-pixel left/right/top/bottom image translation and cropping as well as horizontal flipping (mirroring) (refer to Figure 12), while classes with less than 100 images underwent a process of 5-pixel left/right/top/bottom image translation and cropping as well as horizontal flipping. Sub-databases 1 and 2 from Table 4 were combined to obtain around 600,000 augmented images. In the ChokePoint dataset, unlike DFB-DB1, there were many images for each class, so image translation and cropping was performed at 2-pixel increments in the upper-left direction and 2-pixel increments in the lower-right direction to increase the number of images by a factor of 25. In addition, a horizontal flipping process was performed to increase the number of images by a factor of 50. Sub-databases 1 and 2 from Table 4 were combined to obtain around 740,000 augmented images. This data augmentation method has been used many times in previous studies [76].

**Figure 13:**
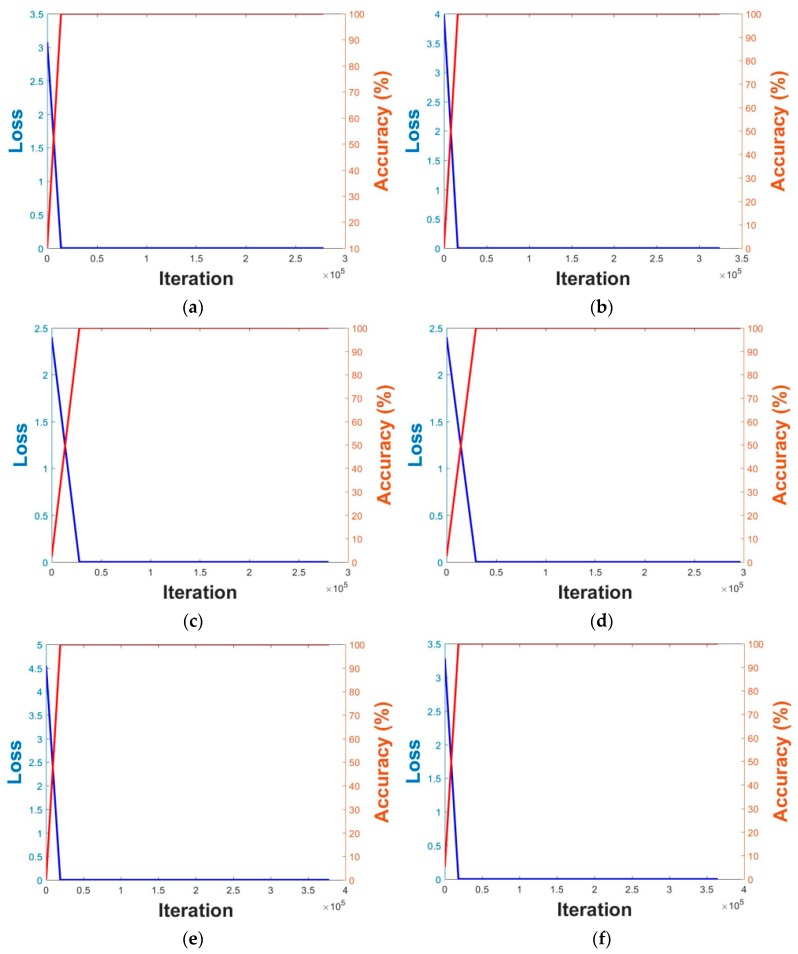

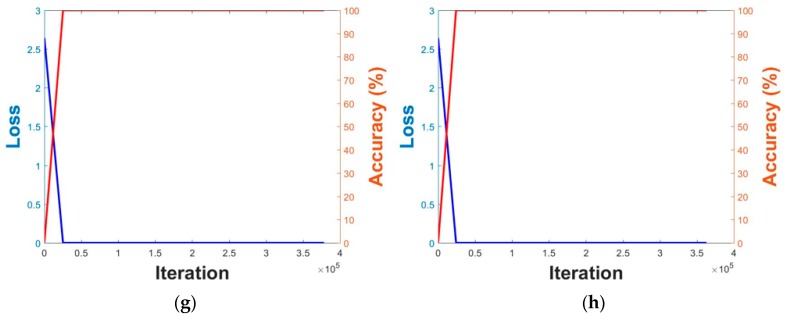
Graphs of training loss and accuracy on DFB-DB1 (**a**–**d**) and ChokePoint datasets (**e**–**h**). VGG Face-16 in case of (**a**,**e**) the 1st validation, (**b**,**f**) the 2nd validation. ResNet-50 in case of (**c**,**g**) the 1st validation, (**d**,**h**) the 2nd validation. Red and blue lines show the training accuracy and loss, respectively.

Using the augmented data, fine-tuning was performed on pre-trained VGG Face-16 and ResNet-50 models using the SGD method. As explained in Section 4.5, unlike the GD method, in the SGD method, the number of training sets divided by mini-batch size is defined as an iteration, and one epoch is set when training is performed for all the iterations. In this study, the momentum, weight decay, and learning rate during training were set at 0.9, 5 × 10^−4^, and 1 × 10^−5^, respectively, and the batch size was 20. Training with DFB-DB1 was performed for 20 epochs, and training with the ChokePoint database was performed for 15 epochs. Because the number of images in the ChokePoint database is larger than that in DFB-DB1 as shown in Table 4, CNN training with the ChokePoint database was performed by the smaller number of epochs than that in DFB-DB1 considering the limitation of graphic processing unit (GPU) memory. Figure 13 shows the training loss and accuracy during the 1st and 2nd validations using DFB-DB1 and the ChokePoint databases. The x axis shows the number of iterations, while the left-side of the y axis shows the loss value and the right-side of the y axis shows the training accuracy. As seen in Figure 13, the training loss was close to 0%, and the training accuracy was close to 100% in all cases. This indicates that the VGG Face-16 and ResNet-50 models used in this study were sufficiently trained. Experimental results showed that it took two or three days for training one model in each fold.

### 5.3. Testing of Proposed Method

#### 5.3.1. Comparisons of Accuracy Achieved by VGG Face-16 and ResNet-50 for Face or Body Recognition

The first experiment measured the accuracy of the VGG Face-16 face recognition and the ResNet-50 body recognition. An equal error rate (EER) was found from the authentic and imposter matching distribution, which was based on the Euclidean distance between the enrolled and input images calculated based on the 4096 features of VGG Face-16. Also, an EER was found from the authentic and imposter matching distribution, which was based on the Euclidean distance between the enrolled and input images calculated based on the 2048 features of ResNet-50. Authentic matching occurs when the enrolled and input images are images of the same class, and imposter matching occurs when they are images of different classes. Also, an error in which an authentic match is incorrectly rejected as an imposter match is called a false rejection error (FRR). Conversely, an error in which an imposter match is incorrectly accepted as an authentic match is called a false acceptance error (FAR). FRR and FAR have a trade-off relationship with each other, and the point at which the FAR and FRR rates become the same is called the equal error rate (EER). As mentioned earlier, experiments were performed with two-fold cross validation using the mean error obtained from testing two times.

First, to compare the recognition accuracy of each CNN model in the face and body regions, the EER of VGG Face-16 and ResNet-50 in testing after training was measured for facial recognition and body recognition, as shown in Table 5 and Table 6, respectively. As seen in the tables, VGG Face-16 made fewer errors in face recognition, and ResNet-50 made fewer errors in body recognition. This suggests that ResNet-50, which has more layers and uses detailed residual information, showed better performance in the body region because the body region is larger than the face region and detailed texture, color, and shape data must be extracted from the clothes and body. Conversely, VGG Face-16 showed better performance than ResNet-50 in the face region because the face region is smaller than the body region, and normally mid- or low-frequency information is used in recognition rather than high-frequency information.

#### 5.3.2. Comparisons of Accuracy Achieved by Single Modality-Based Method and Score-Level Fusions

In the next experiment, the accuracy of single modality-based recognition, which uses face and human recognition using body individually, was compared with the accuracy of the score-level fusion used in this study. For score-level fusion, the weighted sum and weighted product methods described in Section 4.6 were compared. As seen in Table 7 and Table 8, the weighted sum method achieved higher accuracy than the weighted product method in both databases, and it achieved higher accuracy than single modality-based recognition of the face and body without score-level fusion. That is because the two dimensional classifier based the two scores of face and human recognition using body is used for classification in case of score-level fusion whereas one dimensional classifier is used for single modality-based recognition.

**Figure 14:**
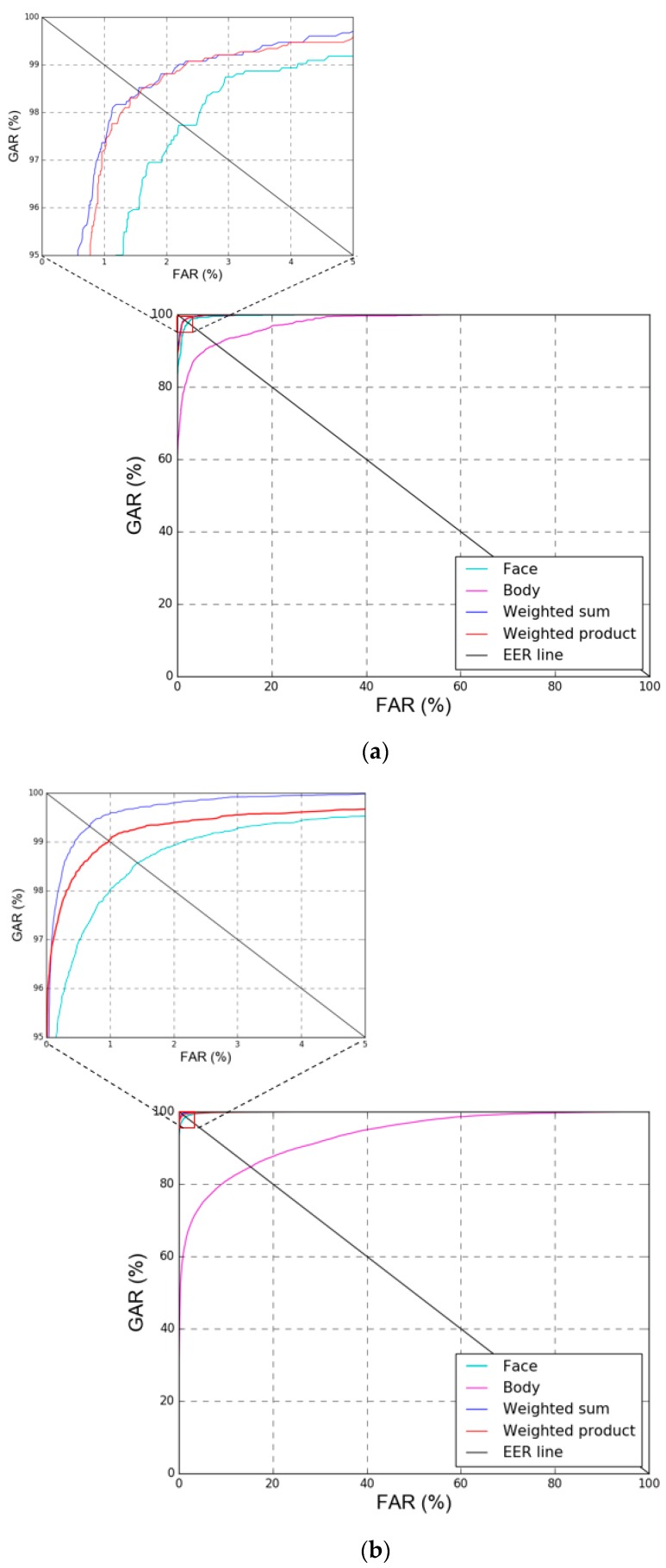
ROC curves by single modality-based method and score-level fusion. With (**a**) DFB-DB1, (**b**) ChokePoint dataset. GAR, FAR, and EER mean genuine acceptance rate, false acceptance rate, and equal error rate, respectively.

Figure 14 shows the receiver operating characteristic (ROC) curves [77] of the results of Table 7 and Table 8. Here, the genuine acceptance rate (GAR) is defined as 100-FRR (%). As previously mentioned, the experiments in this study were performed with two-fold cross validation, and the average graph of the ROC curve obtained from testing two times is shown. In Figure 14, it can be seen that the weighted sum method showed higher accuracy than the weighted product method in both databases, and it showed higher accuracy than single modality-based recognition of the face and body without score-level fusion.

#### 5.3.3. Cases of Correct Recognition, False Acceptance (FA), and False Rejection (FR)

**Figure 15:**
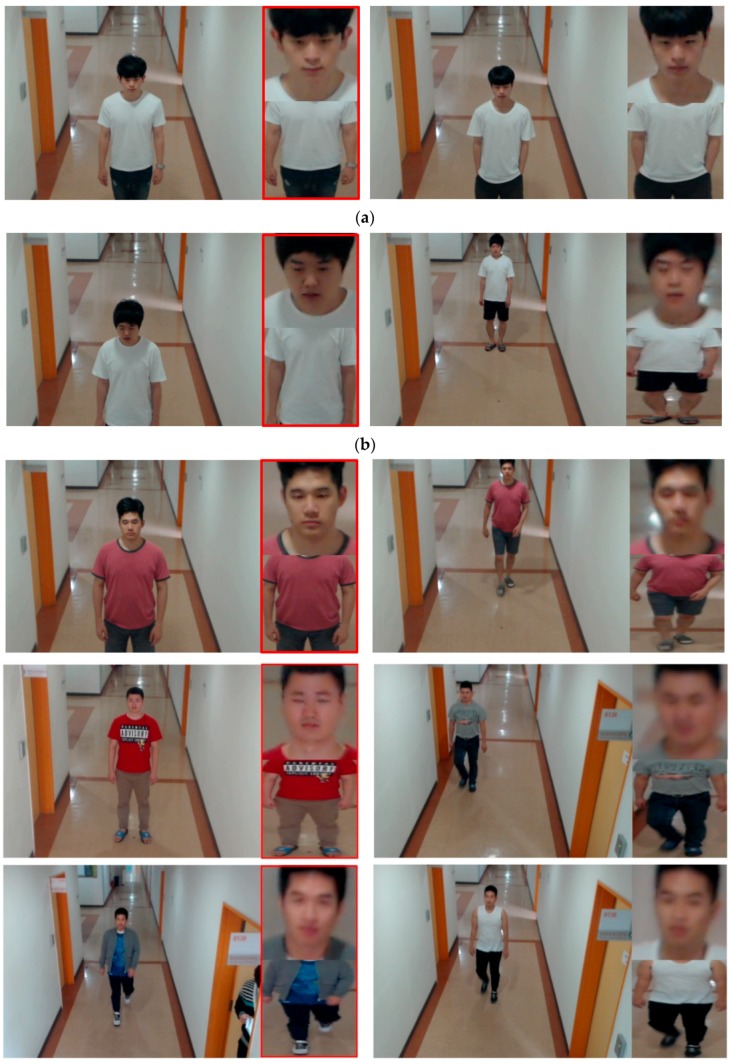

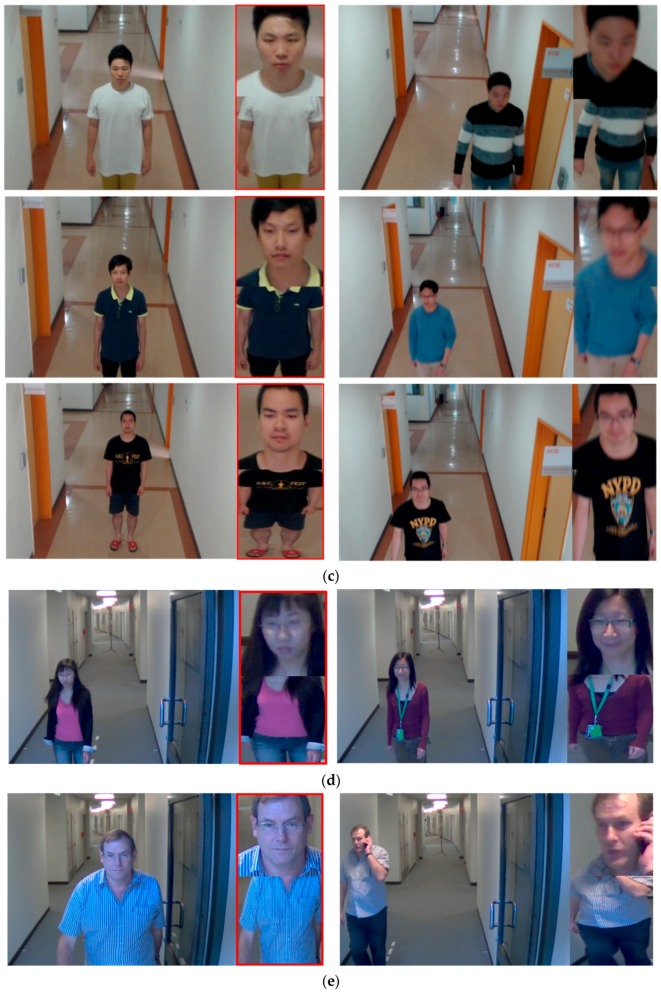

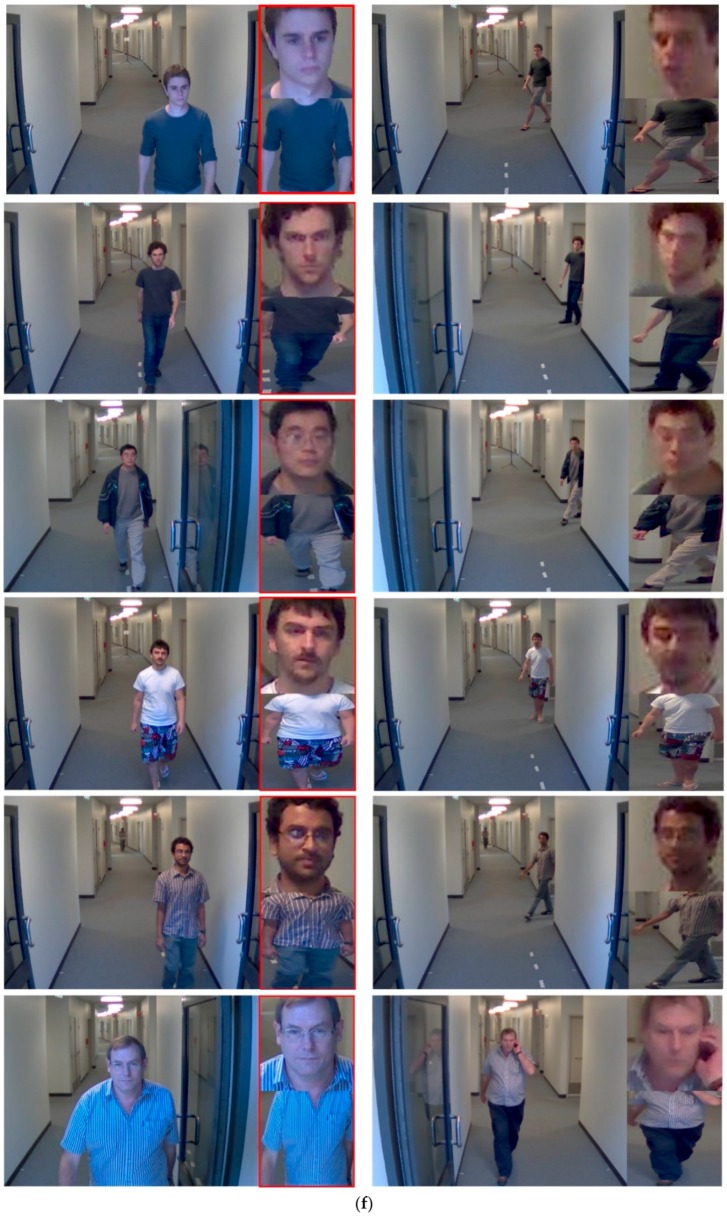
Cases of false acceptance (FA), false rejection (FR), and correct recognition. (**a**–**c**) cases from Dongguk face and body database (DFB-DB1), (**d**–**f**) cases from ChokePoint dataset. (**a**,**d**) FA cases. (**b**,**e**) FR cases. (**c**,**f**) cases of correct recognition.

In this section, we present cases of correct recognition, false acceptance, and false rejection as shown in Figure 15. The image in the red box on the left side of Figure 15 is the enrolled image, and the image on the right side is the recognition image.

As seen in Figure 15a,d, FA occurred when the face and body shapes were similar even thought it was an imposter. Also, as shown in Figure 15b,e, FR occurred when the face was blurred, when a hand and mobile phone were partially included in the face region, when changes in the face pose occurred, and when there was a big difference in the body shape between the enrolled image and the recognition image (when legs were only included in the recognition image and changes in the body’s pose had occurred). However, as Figure 15c demonstrates, even when there was face blurring, the difference in body shape and size between the enrolled image and the recognition image, correct recognition results were achieved by the method proposed in this study. As shown in the 2nd to 6th row images of Figure 15c, the same people even with different clothes were correctly recognized by our system. That is because the person’s whole body image is not used as a single CNN input. Rather, the face region and the body region are separated, and each is used as a separate CNN input. Therefore, the difference of clothes can be compensated by face recognition. In particular, if we disregard the case shown in Figure 15f, where the recognition image is captured at a long distance at the moment the person is coming around a corner and the face image’s resolution is very poor and there are large changes in body shape and pose, the correct recognition results were achieved through score-level fusion of the 2 deep CNN results that were used in this study.

#### 5.3.4. Comparison of Recognition Accuracy by Proposed Method and Using One CNN Based on Full Body Image, and That with and without Data Augmentation

In the next experiment, a performance comparison was made between the method proposed in this study, in which face and body regions are separately processed by two CNNs and score-level fusion is performed, and a method which performs recognition based on one CNN that uses the face and body regions in a single input image. For experiments, VGG Face-16 and ResNet-50 models were fine-tuned with our experimental images. As seen in Table 9, the method proposed in this study achieved higher recognition accuracy. It was possible to use the method to recognize more detailed texture, color, and shape data in each region by separating the face and body regions and using them as input in separate CNNs.

As the next experiment, we compared the accuracy of the models with and without data augmentations. For fair comparison, same procedure of two-fold cross validation was adopted for both methods with and without data augmentations. As shown in Table 10, the EER of recognition with data augmentation is much lower than that without augmentation. The reason why the EER becomes higher without data augmentation is that the number of data is insufficient for training our deep CNN.

As the next experiment, we included the analysis of the influence of focus assessment on the next steps of proposed method. For that, we performed the additional experiments to measure the recognition accuracies with and without focus assessment. For fair comparison, same procedure of two-fold cross validation was adopted for both methods with and without focus assessment. As shown in Table 11, our method with focus assessment shows much lower errors of recognition compared to that without focus assessment. Without focus assessment, severely blurred images are attempted to be recognized, which increases the errors of recognition.

#### 5.3.5. Comparisons of Accuracies by Proposed and Previous Methods

The next experiment compared the recognition accuracy of the proposed method and that of previous methods based on HOG [52] and multi-level local binary pattern (MLBP) + PCA [50,51]. When the accuracy of previous methods was assessed, the methods were divided into two types according to the way of determining enrolled images, and the experiments were performed. 

**Figure 16:**
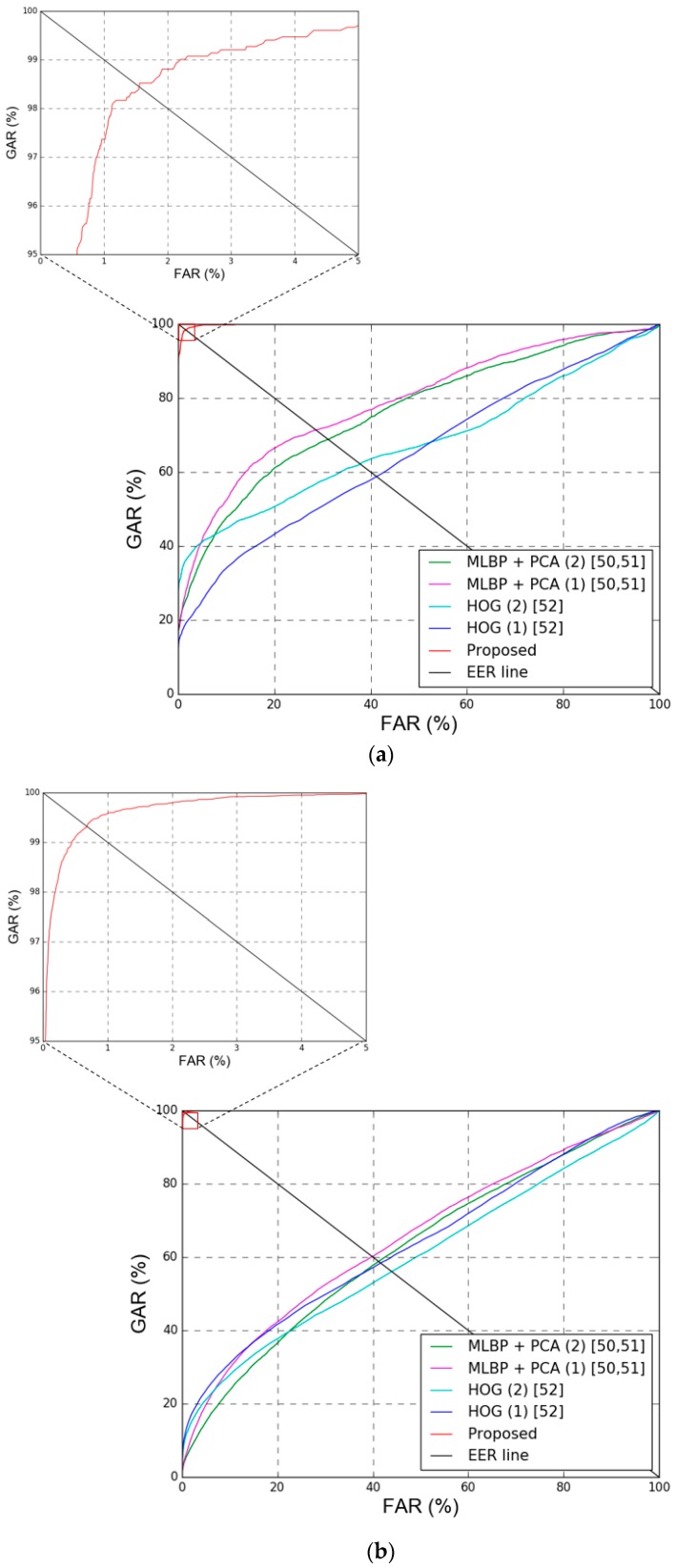
ROC curves by proposed and previous methods. With (**a**) Dongguk face and body database (DFB-DB1) and (**b**) ChokePoint datasets. In (**a**,**b**), multi-level local binary pattern (MLBP) + principal component analysis (PCA) (1) and MLBP + PCA (2) mean the methods of MLBP + PCA based on geometric center by feature difference and pixel difference of Table 12, respectively. In addition, histogram of oriented gradient (HOG) (1) and HOG (2) mean the methods of HOG based on geometric center by feature difference and pixel difference of Table 12, respectively. GAR, FAR, and EER mean genuine acceptance rate, false acceptance rate, and equal error rate, respectively.

The first type determines enrolled images by assuming that the image with the smallest mean value for the image pixel difference with different images in the same class is the geometric center of the feature space. The second type determines enrolled images by assuming that the image with the smallest mean value for the feature difference with different images in the same class is the geometric center of the feature space. For fair comparison, same procedure of two-fold cross validation was adopted for all the experiments. As shown in Table 12 and Figure 16, the other methods all have lower recognition accuracy than the proposed method.

**Figure 17:**
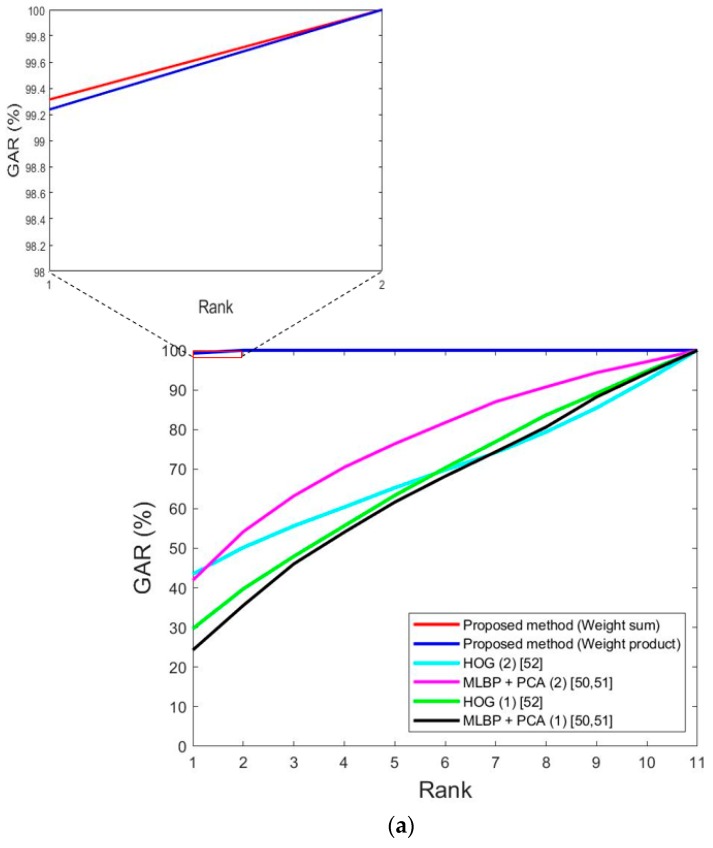

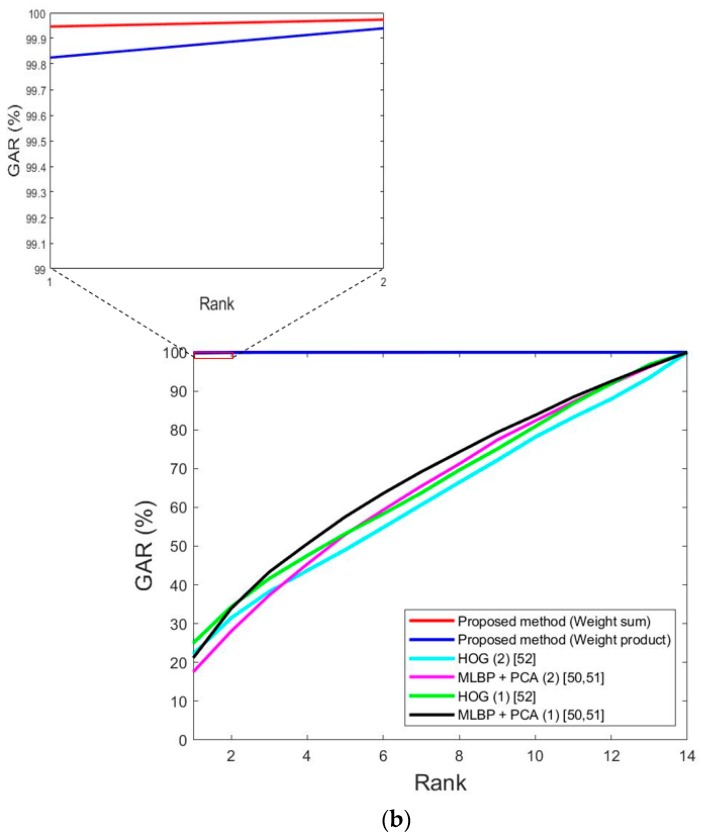
CMC curves by proposed and previous methods. With (**a**) Dongguk face and body database (DFB-DB1) and (**b**) ChokePoint dataset. In (**a**,**b**), multi-level local binary pattern (MLBP) + principal component analysis (PCA) (1) and MLBP + PCA (2) mean the methods of MLBP + PCA based on geometric center by feature difference and pixel difference of Table 12, respectively. In addition, histogram of oriented gradient (HOG) (1) and HOG (2) mean the methods of HOG based on geometric center by feature difference and pixel difference of Table 12, respectively.

The next experiment measured the cumulative match characteristic (CMC) curve to evaluate identification accuracy. Figure 17 shows the CMC curves. The horizontal axis shows the rank, and the vertical axis shows the accuracy (GAR) by rank. As shown in Table 4, 11 people’s data are included in both sub-datasets 1 and 2 for DFB-DB1, and the maximum rank becomes 11 as shown in Figure 17a. In addition, as shown in Table 4, 14 people’s data are included in both sub-datasets 1 and 2 for ChokePoint datasets, and the maximum rank becomes 14 as shown in Figure 17b. As an example, the meaning of a 90% GAR at rank 10 is that when the enrolled image with the smallest matching distance to the input image is selected, the case where the selected image is included in the 10 candidates based on matching distance rank is considered a genuine acceptance case, and the accuracy of this is 90%. 

As shown in Table 4, there were 11 people in the DFB-DB1 database’s testing sub-database and 14 people in the ChokePoint database’s testing sub-database, so the horizontal axes in Figure 17a,b show 11 and 14. As seen in Figure 17, the accuracy of the proposed method was higher than that of previous methods in terms of the CMC curves.

#### 5.3.6. Discussion

Gait recognition with continuous images can show better accuracy than our single-image based approach combining face and body recognition. However, in most previous researches for gait recognition with continuous images [78,79,80,81,82,83,84,85], the accurate region and boundary of human body including the legs should be segmented by correct image binarization in advance. This is because GEI-based methods have been widely used in gait recognition, and they are based on the accumulated binarized image of human body in successive images. For that, the body geometric centers of successive images should be accurately aligned in order to obtain the correct movement information of human gait. If the segmented region of human body is not accurate, the calculated geometric center is not correct, either, which causes the extraction of incorrect movement information of human gait and consequent recognition error increases. In addition, the noise regions connected to the segmented human legs can causes the decrease of recognition accuracy. However, the accurate segmentation of human body including legs is difficult task requiring much processing time in the multiple and continuous images by visible light camera of surveillance environments due to the various environmental factors such as the variations of illuminations and shadows, etc. In addition, it is often the case that the leg parts of human (which are essential information for conventional gait recognition [78,79,80,81,82,83,84,85]) are not visible in our experimental images as shown in Figure 9, Figure 10, Figure 11 and Figure 15.

However, we use the roughly detected region of body in a single image as shown in Figure 2d for recognition without the accurate segmentation of human body region and the alignment of body geometric center. It reduces the processing complexity and the performance of our system can be less affected by the detection accuracy of body regions. Even in the case that legs are not visible in the captured image, our method can correctly recognize human as shown in the 1st, 4th, 5th, 6th row images of Figure 15c and the 1st and 6th row images of Figure 15f.

As shown in Table 13, we compared the processing speed by our method with that by gait-based method [78]. Experimental platform is explained at the end of Section 5.1. As explained, the accurate segmentation of human body is important. However, experimental result showed that the segmentation performance based on background subtraction was bad with our experimental database due to the various factors of illumination variation and shadow, etc. Therefore, we adopted the deep learning-based segmentation method [86] for body segmentation, which was fine-tuned with our experimental database. As shown in Table 13, the processing speed per an image by our method is much faster than that by previous method.

In future, we are planning a study to improve recognition performance by automatically recreating the parts of the body region that cannot be seen in the images using a generative adversarial network (GAN). In addition, we plan to improve recognition performance by using super-resolution reconstruction to restore long-distance low-resolution images and make them into high-resolution images.

## 6. Conclusions

This paper proposed a multimodal human recognition method that uses both the face and body regions in indoor surveillance camera environments, and is based on deep CNNs (VGG Face-16 CNN and ResNet-50 CNN) by score-level fusion of Weighted Sum rule. Unlike previous methods, the proposed method recognizes the face and body regions in a single image separately and combines them to perform recognition in cases where the subject is approaching or moving further away from the camera, which occur frequently in an indoor surveillance camera environment (particularly hallways). In addition, whole body images of people are not used as input for a CNN. Instead, the face and body regions are separated and used as input for separate CNNs. Thus, the system can be used to recognize more detailed texture, color, and shape data for each region, and consequently, it can achieve better recognition accuracy than methods that use a whole body image as input for a single CNN. Unlike previous methods that focus only on cases where the entire body is included in the input images, the proposed method performs recognition on images where part of the body cannot be seen in the input images. To make impartial comparison experiments possible, we have publicly released [53] the VGG Face-16 and ResNet-50 CNN models which were trained in this study, along with the DFB-DB1 database which was custom made using two kinds of cameras to evaluate the performance of the proposed method in a variety of camera environments. In performance evaluations based on EER, ROC curves and CMC curves, it was confirmed that the proposed method (the EERs of 1.52% for DFB-DB1 and 0.58% for the ChokePoint dataset, and the GARs of rank1 of about 99.3% for DFB-DB1 and 99.95% for the ChokePoint dataset) is superior in comparison to face or body single modality-based recognition and other methods used in previous studies. However, FA and FR occurred in cases in which there was a big shape change between the enrolled images and the recognition image (particularly when part of the body could not be seen), as well as cases in which the image was captured at a long distance and had very poor resolution and cases in which there were large changes in the person’s pose between images.

## Figures and Tables

**Table 1:** Summary of our study and previous works on human recognition.

Category		Method	Advantage	Disadvantage
Single modality-based	Face recognition	ASM and image morphing [15]	Not affected by changes in people’s clothes, etc. In comparison to body recognition, few cases occur where part of the region is not captured or pose variation happens.	Difficult to capture front face images. Face frontalization is difficult due to motion and optical blurring in the captured face images.
		DBN [16]		
		PCA [17]		
		SML-MKFC with DA [18]		
		ResNet [40,41]		
	Texture-, color-, and shape-based body recognition using single frame	AlexNet-CNN, HOG, and Mini-CNN[20], VGG [42,43]	Using body information, which has a larger area than the face, and recognition at long distances is possible.	Can misidentify an imposter as being the genuine person if they wear the same clothes. Reduced recognition performance in case that part of the target body is not captured.
		SDALF + MLA [21]		
		CNN + PCA [14]		
		HOG + PCA + SVM [22]		
		Semi-supervised MFL [23]		
		Spatial covariance region [24]		
		SCSP + SPM [26]		
		FPNN [28]		
		S-CNN [29,30]		
		CNN + DDML [31]		
		Multi-level descriptor by WLC [32]		
		LOMO + XQDA [27]		
		Ensemble ranking SVM [25]		
	Body movement (gait)-based recognition using multiple frames	PCA + silhouette analysis-based gait recognition [33]	Higher recognition accuracy than body recognition based on a single image.	Difficult to use when a person approaches or moves further away from the camera. By processing continuous images, the processing time is long.
		Synthetic GEI, PCA + MDA [34]		
Multiple modality-based	Side face recognition + body movement (gait)-based recognition using multiple frames	ESFI + GEI [35,36]	Higher recognition accuracy than single modality-based methods for face recognition or body movement-based recognition.	
		Side face + GEI [37]		
		Curvature-based matching + direct GEI [38]		
		Posterior distribution + template matching [39]		
		Image-based VH [44]		
		View-normalized sequences [45]		
		KFA + RSM framework [46]		
		Eigenface calculation +α-GEI [47]		
		HMM + Gabor-based EBGM [48]		
		Fisherface + silhouette image-based LPP [49]		
	Frontal face and texture-, color-, and shape-based body recognition using single frame	MLBP + PCA [50,51], HOG [52]	**–** Higher recognition accuracy than single modality-based methods **–** By single image processing, processing speed is fast.	Lower accuracy than deep CNN-Based method
		Deep CNN-based multimodal human recognition using both face and body (Proposed method)		Requiring an intensive training process of CNN

**Table 2:** Descriptions of VGG Face-16 model.

Layer Type		Number of Filters	Size of Feature Map	Size of Filter	Number of Strides	Amount of Padding
Image input layer			224 (height) × 224 (width) × 3 (channel)			
Group 1	Conv1_1 (1st convolutional layer)	64	224 × 224 × 64	3 × 3	1 × 1	1 × 1
	ReLU1_1		224 × 224 × 64			
	Conv1_2 (2nd convolutional layer)	64	224 × 224 × 64	3 × 3	1 × 1	1 × 1
	ReLU1_2		224 × 224 × 64			
	MaxPool1	1	112 × 112 × 64	2 × 2	2 × 2	0 × 0
Group 2	Conv2_1 (3rd convolutional layer)	128	112 × 112 × 128	3 × 3	1 × 1	1 × 1
	ReLU2_1		112 × 112 × 128			
	Conv2_2 (4th convolutional layer)	128	112 × 112 × 128	3 × 3	1 × 1	1 × 1
	ReLU2_2		112 × 112 × 128			
	MaxPool2	1	56 × 56 × 128	2 × 2	2 × 2	0 × 0
Group 3	Conv3_1 (5th convolutional layer)	256	56 × 56 × 256	3 × 3	1 × 1	1 × 1
	ReLU3_1		56 × 56 × 256			
	Conv3_2 (6th convolutional layer)	256	56 × 56 × 256	3 × 3	1 × 1	1 × 1
	ReLU3_2		56 × 56 × 256			
	Conv3_3 (7th convolutional layer)	256	56 × 56 × 256	3 × 3	1 × 1	1 × 1
	ReLU3_3		56 × 56 × 256			
	MaxPool3	1	28 × 28 × 256	2 × 2	2 × 2	0 × 0
Group 4	Conv4_1 (8th convolutional layer)	512	28 × 28 × 512	3 × 3	1 × 1	1 × 1
	ReLU4_1		28 × 28 × 512			
	Conv4_2 (9th convolutional layer)	512	28 × 28 × 512	3 × 3	1 × 1	1 × 1
	ReLU4_2		28 × 28 × 512			
	Conv4_3 (10th convolutional layer)	512	28 × 28 × 512	3 × 3	1 × 1	1 × 1
	ReLU4_3		28 × 28 × 512			
	MaxPool4	1	14 × 14 × 512	2 × 2	2 × 2	0 × 0
Group 5	Conv5_1 (11th convolutional layer)	512	14 × 14 × 512	3 × 3	1 × 1	1 × 1
	ReLU5_1		14 × 14 × 512			
	Conv5_2 (12th convolutional layer)	512	14 × 14 × 512	3 × 3	1 × 1	1 × 1
	ReLU5_2		14 × 14 × 512			
	Conv5_3 (13th convolutional layer)	512	14 × 14 × 512	3 × 3	1 × 1	1 × 1
	ReLU5_3		14 × 14 × 512			
	MaxPool5	1	7 × 7 × 512	2 × 2	2 × 2	0 × 0
Fc6 (1st fully connected layer)			4096 × 1			
ReLU6			4096 × 1			
Dropout6			4096 × 1			
Fc7 (2nd fully connected layer)			4096 × 1			
ReLU7			4096 × 1			
Dropout7			4096 × 1			
Fc8(3rd fully connected layer)			#classes			
Softmax layer			#classes			
Output layer			#classes			

**Table 3:** Output size, numbers and sizes of filters, number of strides, and amount of padding in our deep residual CNN structure (3* indicates that 3 pixels are included as padding in left, right, up, and down positions of input image of 224 × 224 × 3, whereas 1* indicates that 1 pixel is included as padding in left, right, up, and down positions of feature map) (2/1** indicates 2 at the 1st iteration and 1 at the 2nd iteration) (For the shortcuts in Conv2_4, 3_4, 4_4, and 5_4, the filter of 1 × 1 is used only for the 1st iteration whereas identity mapping is used for the other iterations).

Layer Type		Size of Feature Map	Number of Filters	Size of Filters	Number of Strides	Amount of Padding	Number of Iterations
Image input layer		224 (height) × 224 (width) × 3 (channel)					
Conv1		112 × 112 × 64	64	7 × 7	2	3*	1
Max pool		56 × 56 × 64	1	3 × 3	2	0	1
Conv2	Conv2_1	56 × 56 × 64	64	1 × 1	1	0	3
	Conv2_2	56 × 56 × 64	64	3 × 3	1	1*	
	Conv2_3	56 × 56 × 256	256	1 × 1	1	0	
	Conv2_4 (Shortcut)	56 × 56 × 256	256	1 × 1	1	0	
Conv3	Conv3_1	28 × 28 × 128	128	1 × 1	2/1**	0	4
	Conv3_2 (Bottleneck)	28 × 28 × 128	128	3 × 3	1	1*	
	Conv3_3	28 × 28 × 512	512	1 × 1	1	0	
	Conv3_4 (Shortcut)	28 × 28 × 512	512	1 × 1	2	0	
Conv4	Conv4_1	14 × 14 × 256	256	1 × 1	2/1**	0	6
	Conv4_2 (Bottleneck)	14 × 14 × 256	256	3 × 3	1	1*	
	Conv4_3	14 × 14 × 1024	1024	1 × 1	1	0	
	Conv4_4 (Shortcut)	14 × 14 × 1024	1024	1 × 1	2	0	
Conv5	Conv5_1	7 × 7 × 512	512	1 × 1	2/1**	0	3
	Conv5_2 (Bottleneck)	7 × 7 × 512	512	3 × 3	1	1*	
	Conv5_3	7 × 7 × 2048	2048	1 × 1	1	0	
	Conv5_4 (Shortcut)	7 × 7 × 2048	2048	1 × 1	2	0	
AVG pool		1 × 1 × 2048	1	7 × 7	1	0	1
FC layer		2					1
Softmax		2					1

**Table 4:** Descriptions of DFB-DB1 and ChokePoint dataset.

		Face		Body	
		Sub-Dataset 1	Sub-Dataset 1	Sub-Dataset 1	Sub-Dataset 1
DFB-DB1	Number of people	11	11	11	11
	Number of images	564	767	564	767
	Number of augmented images (for training)	278,300	324,038	278,300	324,038
ChokePoint dataset	Number of people	14	14	14	14
	Number of images	7565	7296	7565	7296
	Number of augmented images (for training)	378,250	364,800	378,250	364,800

**Table 5:** Comparisons of EERs by VGG Face-16 and ResNet-50 for face recognition (unit: %).

	VGG Face-16	ResNet-50 [40,41]
1st fold	2.03	9.11
2nd fold	2.49	17.7
Average	2.26	13.405

**Table 6:** Comparisons of EERs by VGG Net-19 and ResNet-50 for body recognition (unit: %).

	VGG Net-19 [42,43]	ResNet-50
1st fold	27.52	8.82
2nd fold	16.21	7.88
Average	21.865	8.35

**Table 7:** Comparisons of EERs by face and human recognition using body (unit: %).

Modality	DFB-DB1			ChokePoint Dataset		
	1st Fold	2nd Fold	Average	1st fold	2nd Fold	Average
Face	2.03	2.49	2.26	1.49	1.38	1.435
Body	8.82	7.88	8.35	18.44	10.67	14.56

**Table 8:** Comparisons of EER by score-level fusion (unit: %).

Method	DFB-DB1			ChokePoint Dataset		
	1st Fold	2nd Fold	Average	1st Fold	2nd Fold	Average
Weighted Sum	0.9	2.13	1.52	0.37	0.79	0.58
Weighted Product	0.92	2.23	1.58	1.12	0.88	1

**Table 9:** Comparisons of EERs by proposed method and using one CNN based on full body image (unit: %).

	Using One CNN Based on Full Body Image (VGG Face-16) [42]	Using One CNN Based on Full Body Image (ResNet-50)	Proposed Method
1st fold	12.49	4.98	0.9
2nd fold	13.59	2.65	2.13
Average	13.04	3.815	1.52

**Table 10:** Comparisons of EERs with and without data augmentation (unit: %).

	With Augmentation			Without Augmentation		
	Face	Body	Combined	Face	Body	Combined
1st fold	2.03	8.82	0.9	13.56	50.32	13.53
2nd fold	2.49	7.88	2.13	12.6	17.24	10.3
Average	2.26	8.35	1.52	13.08	33.78	11.92

**Table 11:** Comparisons of EERs with and without focus assessment (unit: %).

	With Focus Assessment			Without Focus Assessment		
	Face	Body	Combined	Face	Body	Combined
1st fold	2.03	8.82	0.9	47.19	32.58	29.67
2nd fold	2.49	7.88	2.13	47.09	28.94	26.42
Average	2.26	8.35	1.52	47.14	30.76	28.05

**Table 12:** Comparison of EERs by proposed and previous methods (unit: %).

Method		DFB-DB1			ChokePoint Dataset		
		1st Fold	2nd Fold	Average	1st Fold	2nd Fold	Average
HOG [52]	Geometric center by pixel difference	40.13	35.67	37.9	45.98	42.19	44.09
	Geometric center by feature difference	38.09	44.14	41.12	41.84	41.47	41.66
MLBP + PCA [50,51]	Geometric center by pixel difference	31.72	30.62	31.17	41.92	39.9	40.91
	Geometric center by feature difference	29.38	27.84	28.61	37.75	42.38	40.07
Proposed method		0.9	2.13	1.52	0.37	0.79	0.58

**Table 13:** Comparison of processing time per an image by proposed and previous method (unit: ms).

**Method**	**Body Segmentation & Alignment**	**Matching Based on Radon Transform and PCA**	**Total**
Gait-based method [78]	752	145	897
**Method**	**Face & body detection**	**Matching based on two CNNs**	**Total**
Proposed method	98	327	425
